# Supplementary material for: A Transcriptomic Response to Lactiplantibacillus plantarum-KCC48 against High-Fat Diet-Induced Fatty Liver Diseases in Mice
Source: Int J Mol Sci. 2022 Jun 17;23(12):6750. doi: 10.3390/ijms23126750 (PMC9224190; doi:10.3390/ijms23126750)
Supplement: Supplementary file 1 [file ijms-23-06750-s001.zip › ijms-1745422-supplementary.pdf]

Article

A Transcriptomic Response to *Lactiplantibacillus plantarum*-KCC48 against High-Fat Diet-Induced Fatty Liver Diseases in Mice

Ilavenil Soundharrajan [1], Muthusamy Karnan [1], Jeong-Sung Jung [1], Kyung-Dong Lee [2], Jeong-Chae Lee [3], Thiyagarajan Ramesh [4], Dahye Kim [5,*] and Ki-Choon Choi [1,*]

[1] Grassland and Forage Division, Rural Development Administration, National Institute of Animal Science, Cheonan 31000, Korea; ilavenil@korea.kr (I.S.); karnan7899@rediffmail.com (M.K.); jjs3873@korea.kr (J.-S.J.)

[2] Department of Companion Animals, Dongsin University, Naju 58245, Korea; leekd@dsu.ac.kr

[3] Department of Bioactive Material Sciences and Research Center of Bioactive Materials, Jeonbuk National University, Jeonju 54896, Korea; leejc88@jbnu.ac.kr

[4] Department of Basic Medical Sciences, College of Medicine, Prince Sattam Bin Abdulaziz University, Al-Kharj 11942, Saudi Arabia; thiyagaramesh@gmail.com

[5] Animal Genomics and Bioinformatics Division, National Institute of Animal Science, Wanju 55365, Korea

* Correspondence: dhkim0724@korea.kr (D.K.); choiwh@korea.kr (K.-C.C.); Tel.: +82-41-580-6752 (D.K.); Fax: +82-41-580-6779 (K.-C.C.)

Table S1. Differentially expressed genes in the livers of HFD-fed animals compared with controls

S. No	Gene Symbol	Gene Name	Fold Changes	*P* value
**1**	1810008I18Rik	RIKEN cDNA 1810008I18 gene(1810008I18Rik)	0.445	0.00005
**2**	9030619P08Rik	lymphocyte antigen 6 complex pseudogene(9030619P08Rik)	2.094	0.00005
**3**	Abcc3	ATP-binding cassette, sub-family C (CFTR/MRP), member 3(Abcc3)	3.739	0.00005
**4**	Acaa1b	acetyl-Coenzyme A acyltransferase 1B(Acaa1b)	2.846	0.00005
**5**	Acadm	acyl-Coenzyme A dehydrogenase, medium chain(Acadm)	2.081	0.00005
**6**	Acly	ATP citrate lyase(Acly)	0.349	0.00005
**7**	Acmsd	amino carboxymuconate semialdehyde decarboxylase(Acmsd)	0.442	0.00005
**8**	Acnat2	acyl-coenzyme A amino acid N-acyltransferase 2(Acnat2)	2.775	0.00005
**9**	Acot1	acyl-CoA thioesterase 1(Acot1)	5.142	0.00005
**10**	Acss2	acyl-CoA synthetase short-chain family member 2(Acss2)	0.484	0.00005
**11**	Acss3	acyl-CoA synthetase short-chain family member 3(Acss3)	4.278	0.00005
**12**	Adam11	a disintegrin and metallopeptidase domain 11(Adam11)	2.318	0.00005
**13**	Adhfe1	alcohol dehydrogenase, iron containing, 1(Adhfe1)	2.557	0.00005
**14**	Aldh1a1	aldehyde dehydrogenase family 1, subfamily A1(Aldh1a1)	2.539	0.00005
**15**	Aldh3a2	aldehyde dehydrogenase family 3, subfamily A2(Aldh3a2)	6.414	0.00005
**16**	Ang	angiogenin, ribonuclease, RNase A family, 5(Ang)	0.498	0.04865
**17**	Angptl4	angiopoietin-like 4(Angptl4)	2.147	0.00005
**18**	Anxa2	annexin A2(Anxa2)	2.888	0.00005
**19**	Apol9a	apolipoprotein L 9a(Apol9a)	2.532	0.00005
**20**	Apol9b	apolipoprotein L 9b(Apol9b)	2.080	0.00005
**21**	Apom	apolipoprotein M(Apom)	0.465	0.00005
**22**	Arntl	aryl hydrocarbon receptor nuclear translocator-like(Arntl)	0.477	0.00005
**23**	Arrdc3	arrestin domain containing 3(Arrdc3)	2.038	0.00005
**24**	Avpr1a	arginine vasopressin receptor 1A(Avpr1a)	0.454	0.00005
**25**	Bche	butyrylcholinesterase(Bche)	2.055	0.00005
**26**	Cd36	CD36 antigen(Cd36)	3.558	0.00005
**27**	Cd74	CD74 antigen (invariant polypeptide of major histocompatibility complex, class II antigen-associated)(Cd74)	2.053	0.00005
**28**	Cdh1	cadherin 1(Cdh1)	0.242	0.00005
**29**	Ces1g	carboxylesterase 1G(Ces1g)	2.228	0.00005
**30**	Ces2a	carboxylesterase 2A(Ces2a)	0.290	0.00005
**31**	Chka	choline kinase alpha(Chka)	0.400	0.00005
**32**	Cry1	cryptochrome 1 (photolyase-like)(Cry1)	0.438	0.00005
**33**	Ctse	cathepsin E(Ctse)	3.918	0.00005
**34**	Cyp2a12	cytochrome P450, family 2, subfamily a, polypeptide 12(Cyp2a12)	0.427	0.00005
**35**	Cyp2b9	cytochrome P450, family 2, subfamily b, polypeptide 9(Cyp2b9)	13.813	0.00005
**36**	Cyp2c37	cytochrome P450, family 2. subfamily c, polypeptide 37(Cyp2c37)	3.299	0.00005
**37**	Cyp2c38	cytochrome P450, family 2, subfamily c, polypeptide 38(Cyp2c38)	6.591	0.00005
**38**	Cyp2c39	cytochrome P450, family 2, subfamily c, polypeptide 39(Cyp2c39)	9.718	0.00005
**39**	Cyp2c44	cytochrome P450, family 2, subfamily c, polypeptide 44(Cyp2c44)	2.282	0.00005
**40**	Cyp2c50	cytochrome P450, family 2, subfamily c, polypeptide 50(Cyp2c50)	2.790	0.00005
**41**	Cyp2c54	cytochrome P450, family 2, subfamily c, polypeptide 54(Cyp2c54)	3.351	0.00005
**42**	Cyp3a11	cytochrome P450, family 3, subfamily a, polypeptide 11(Cyp3a11)	5.499	0.00005
**43**	Cyp3a59	cytochrome P450, family 3, subfamily a, polypeptide 59(Cyp3a59)	4.626	0.00005
**44**	Cyp46a1	cytochrome P450, family 46, subfamily a, polypeptide 1(Cyp46a1)	2.026	0.00005
**45**	Cyp4a10	cytochrome P450, family 4, subfamily a, polypeptide 10(Cyp4a10)	26.691	0.00005
**46**	Cyp4a12b	cytochrome P450, family 4, subfamily a, polypeptide 12B(Cyp4a12b)	2.282	0.00005
**47**	Cyp4a14	cytochrome P450, family 4, subfamily a, polypeptide 14(Cyp4a14)	24.394	0.0001
**48**	Cyp51	cytochrome P450, family 51(Cyp51)	0.454	0.00005
**49**	Cyp7a1	cytochrome P450, family 7, subfamily a, polypeptide 1(Cyp7a1)	4.482	0.00005
**50**	Dbp	D site albumin promoter binding protein(Dbp)	13.345	0.0429

**51**	Ddah1	dimethylarginine dimethylaminohydrolase 1(Ddah1)	2.251	0.00005
**52**	Derl3	Der1-like domain family, member 3(Derl3)	3.311	0.00005
**53**	Dhcr7	7-dehydrocholesterol reductase(Dhcr7)	0.454	0.00005
**54**	Dio1	deiodinase, iodothyronine, type I(Dio1)	3.168	0.00005
**55**	Dmbt1	deleted in malignant brain tumors 1(Dmbt1)	47.186	0.00005
**56**	Ehhadh	enoyl-Coenzyme A, hydratase/3-hydroxyacyl Coenzyme A dehydrogenase(Ehhadh)	2.223	0.00005
**57**	Elovl3	elongation of very long chain fatty acids (FEN1/Elo2, SUR4/Elo3, yeast)-like 3(Elovl3)	0.271	0.00005
**58**	Ephx1	epoxide hydrolase 1, microsomal(Ephx1)	2.992	0.00005
**59**	Fabp2	fatty acid binding protein 2, intestinal(Fabp2)	2.224	0.00005
**60**	Fabp4	fatty acid binding protein 4, adipocyte(Fabp4)	2.079	0.00005
**61**	Fabp5	fatty acid binding protein 5, epidermal(Fabp5)	0.076	0.00005
**62**	Fam198a	family with sequence similarity 198, member A(Fam198a)	0.482	0.00005
**63**	Fam25c	family with sequence similarity 25, member C(Fam25c)	0.168	0.00005
**64**	Fasn	fatty acid synthase(Fasn)	0.354	0.00005
**65**	Fdps	farnesyl diphosphate synthetase(Fdps)	0.346	0.00005
**66**	Fgl1	fibrinogen-like protein 1(Fgl1)	0.470	0.00005
**67**	Fitm1	fat storage-inducing transmembrane protein 1(Fitm1)	5.975	0.00005
**68**	Fkbp5	FK506 binding protein 5(Fkbp5)	2.865	0.00005
**69**	Gadd45g	growth arrest and DNA-damage-inducible 45 gamma(Gadd45g)	0.267	0.00005
**70**	Gas6	growth arrest specific 6(Gas6)	2.145	0.00005
**71**	Gm11127	predicted gene 11127(Gm11127)	2.441	0.00005
**72**	Gm3219	B-cell CLL/lymphoma 7C pseudogene(Gm3219)	0.401	0.00005
**73**	Gm6644	Akr1b3 pseudogene(Gm6644)	0.492	0.00005
**74**	Gpc1	glypican 1(Gpc1)	2.999	0.00005
**75**	Gstm2	glutathione S-transferase, mu 2(Gstm2)	2.275	0.00005
**76**	Gstm3	glutathione S-transferase, mu 3(Gstm3)	3.361	0.00005
**77**	H2-Aa	histocompatibility 2, class II antigen A, alpha(H2-Aa)	2.585	0.00005
**78**	H2-Ab1	histocompatibility 2, class II antigen A, beta 1(H2-Ab1)	2.325	0.00005
**79**	H2-Eb1	histocompatibility 2, class II antigen E beta(H2-Eb1)	3.240	0.00005
**80**	H2-Q5	histocompatibility 2, Q region locus 5(H2-Q5)	0.441	0.00005
**81**	Hes6	hairy and enhancer of split 6(Hes6)	0.459	0.00005
**82**	Hexb	hexosaminidase B(Hexb)	2.459	0.004
**83**	Hpgd	hydroxyprostaglandin dehydrogenase 15 (NAD)(Hpgd)	2.304	0.00005
**84**	Hsd17b11	hydroxysteroid (17-beta) dehydrogenase 11(Hsd17b11)	2.025	0.00005
**85**	Idi1	isopentenyl-diphosphate delta isomerase(Idi1)	0.351	0.00005
**86**	Igfbp2	insulin-like growth factor binding protein 2(Igfbp2)	0.483	0.00005
**87**	Inhba	inhibin beta-A(Inhba)	0.465	0.00005
**88**	Insig1	insulin induced gene 1(Insig1)	0.433	0.00005
**89**	Irs2	insulin receptor substrate 2(Irs2)	0.465	0.00005
**90**	Krt19	keratin 19(Krt19)	4.125	0.00005
**91**	Lcn2	lipocalin 2(Lcn2)	3.362	0.00005
**92**	Lpin1	lipin 1(Lpin1)	2.546	0.00005
**93**	Ly6a	lymphocyte antigen 6 complex, locus A(Ly6a)	8.768	0.00005
**94**	Ly6c1	lymphocyte antigen 6 complex, locus C1(Ly6c1)	8.054	0.00005
**95**	Ly6d	lymphocyte antigen 6 complex, locus D(Ly6d)	5.567	0.00005
**96**	Mfsd2a	major facilitator superfamily domain containing 2A(Mfsd2a)	4.328	0.00005
**97**	Mme	membrane metallo endopeptidase(Mme)	2.384	0.00005
**98**	Moxd1	monooxygenase, DBH-like 1(Moxd1)	0.020	0.00005
**99**	Mt2	metallothionein 2(Mt2)	2.602	0.00005
**100**	Mthfr	5,10-methylenetetrahydrofolate reductase(Mthfr)	0.401	0.00005
**101**	Mup21	major urinary protein 21(Mup21)	0.456	0.00005
**102**	Mvd	mevalonate (diphospho) decarboxylase(Mvd)	0.387	0.00005
**103**	Myl9	myosin, light polypeptide 9, regulatory(Myl9)	2.474	0.00005
**104**	Nfil3	nuclear factor, interleukin 3, regulated(Nfil3)	0.482	0.00005
**105**	Nlrp12	NLR family, pyrin domain containing 12(Nlrp12)	2.060	0.00005
**106**	Nr1d1	nuclear receptor subfamily 1, group D, member 1(Nr1d1)	3.334	0.00005

**107**	Nr1d2	nuclear receptor subfamily 1, group D, member 2(Nr1d2)	2.745	0.00005
**108**	Nsdhl	NAD(P) dependent steroid dehydrogenase-like(Nsdhl)	0.458	0.00005
**109**	Obp2a	odorant binding protein 2A(Obp2a)	7.848	0.00005
**110**	Omd	osteomodulin(Omd)	2.364	0.00005
**111**	Orm2	orosomucoid 2(Orm2)	2.307	0.00005
**112**	Osgin1	oxidative stress induced growth inhibitor 1(Osgin1)	0.448	0.00005
**113**	Pcsk9	proprotein convertase subtilisin/kexin type 9(Pcsk9)	0.343	0.00005
**114**	Pctp	phosphatidylcholine transfer protein(Pctp)	2.636	0.00005
**115**	Pmvk	phosphomevalonate kinase(Pmvk)	0.342	0.00005
**116**	Por	P450 (cytochrome) oxidoreductase(Por)	3.068	0.00005
**117**	Ppard	peroxisome proliferator activator receptor delta(Ppard)	0.226	0.00005
**118**	Rad51b	RAD51 paralog B(Rad51b)	3.079	0.00005
**119**	Rapgef4	Rap guanine nucleotide exchange factor (GEF) 4(Rapgef4)	2.154	0.00005
**120**	Rdh11	retinol dehydrogenase 11(Rdh11)	0.369	0.00005
**121**	Rdh16	retinol dehydrogenase 16(Rdh16)	3.376	0.00005
**122**	Rdh9	retinol dehydrogenase 9(Rdh9)	3.309	0.00005
**123**	Rec114	REC114 meiotic recombination protein(Rec114)	2.086	0.03665
**124**	Retsat	retinol saturase (all trans retinol 13,14 reductase)(Retsat)	2.056	0.00005
**125**	Rgs16	regulator of G-protein signaling 16(Rgs16)	4.933	0.00005
**126**	Rmrp	RNA component of mitochondrial RNAase P(Rmrp)	0.281	0.00005
**127**	Rpph1	ribonuclease P RNA component H1(Rpph1)	0.246	0.00005
**128**	Rprl3	ribonuclease P RNA-like 3(Rprl3)	0.301	0.00085
**129**	Rtn4	reticulon 4(Rtn4)	2.115	0.00005
**130**	Saa3	serum amyloid A 3(Saa3)	3.576	0.00005
**131**	Sh3bgrl2	SH3 domain binding glutamic acid-rich protein like 2(Sh3bgrl2)	2.345	0.00005
**132**	Slc16a7	solute carrier family 16 (monocarboxylic acid transporters), member 7(Slc16a7)	2.132	0.00005
**133**	Slco2a1	solute carrier organic anion transporter family, member 2a1(Slco2a1)	0.459	0.00005
**134**	Snord104	small nucleolar RNA, C/D box 104(Snord104)	0.000	0.0001
**135**	Tagln	transgelin(Tagln)	2.419	0.01895
**136**	Tceal8	transcription elongation factor A (SII)-like 8(Tceal8)	2.259	0.00005
**137**	Tef	thyrotroph embryonic factor(Tef)	3.055	0.00005
**138**	Tff3	trefoil factor 3, intestinal(Tff3)	0.135	0.0061
**139**	Tm4sf4	transmembrane 4 superfamily member 4(Tm4sf4)	2.699	0.00005
**140**	Tppp	tubulin polymerization promoting protein(Tppp)	2.242	0.00005
**141**	Tsku	tsukushi, small leucine rich proteoglycan(Tsku)	3.061	0.00005
**142**	Tstd1	thiosulfate sulfurtransferase (rhodanese)-like domain containing 1(Tstd1)	0.499	0.00005
**143**	Ugt1a1	UDP glucuronosyltransferase 1 family, polypeptide A1(Ugt1a1)	2.205	0.01135
**144**	Upp2	uridine phosphorylase 2(Upp2)	5.374	0.00005
**145**	Vnn3	vanin 3(Vnn3)	2.523	0.00005

Table S2. Differences in liver gene expression in animals fed HFLPD compared to HFD alone

S. No	Gene Symbol	Gene Name	Fold Changes	*P* value
**1**	9030619P08Rik	lymphocyte antigen 6 complex pseudogene(9030619P08Rik)	0.493	0.00005
**2**	Abcc3	ATP-binding cassette, sub-family C (CFTR/MRP), member 3(Abcc3)	0.373	0.00005
**3**	Alas1	aminolevulinic acid synthase 1(Alas1)	0.460	0.00005
**4**	Aldh3a2	aldehyde dehydrogenase family 3, subfamily A2(Aldh3a2)	0.448	0.00005
**5**	Avpr1a	arginine vasopressin receptor 1A(Avpr1a)	2.216	0.00005
**6**	BC002163	NADH dehydrogenase Fe-S protein 5 pseudogene(BC002163)	10.423	0.04860
**7**	Capn8	calpain 8(Capn8)	2.402	0.00005
**8**	Cck	cholecystokinin(Cck)	2.269	0.00005
**9**	Cd36	CD36 antigen(Cd36)	0.375	0.00005
**10**	Ces1g	carboxylesterase 1G(Ces1g)	0.356	0.00005
**11**	Cml5	Calmodulin-like protein 5	2.544	0.00005
**12**	Ctse	cathepsin E(Ctse)	0.382	0.00005
**13**	Cwc22	CWC22 spliceosome-associated protein(Cwc22)	0.237	0.00005
**14**	Cyp2a12	cytochrome P450, family 2, subfamily a, polypeptide 12(Cyp2a12)	2.902	0.00005
**15**	Cyp2a5	cytochrome P450, family 2, subfamily a, polypeptide 5(Cyp2a5)	2.061	0.00005
**16**	Cyp2b13	cytochrome P450, family 2, subfamily b, polypeptide 13(Cyp2b13)	0.201	0.00005
**17**	Cyp2b9	cytochrome P450, family 2, subfamily b, polypeptide 9(Cyp2b9)	0.351	0.00005
**18**	Cyp2c38	cytochrome P450, family 2, subfamily c, polypeptide 38(Cyp2c38)	0.273	0.00005
**19**	Cyp2c39	cytochrome P450, family 2, subfamily c, polypeptide 39(Cyp2c39)	0.243	0.00005
**20**	Cyp3a11	cytochrome P450, family 3, subfamily a, polypeptide 11(Cyp3a11)	0.460	0.00005
**21**	Cyp3a59	cytochrome P450, family 3, subfamily a, polypeptide 59(Cyp3a59)	0.397	0.00005
**22**	Cyp4a10	cytochrome P450, family 4, subfamily a, polypeptide 10(Cyp4a10)	0.186	0.00005
**23**	Cyp4a12b	cytochrome P450, family 4, subfamily a, polypeptide 12B(Cyp4a12b)	0.456	0.00005
**24**	Cyp4a14	cytochrome P450, family 4, subfamily a, polypeptide 14(Cyp4a14)	0.180	0.00005
**25**	Cyp7a1	cytochrome P450, family 7, subfamily a, polypeptide 1(Cyp7a1)	0.454	0.00005
**26**	Dbp	D site albumin promoter binding protein(Dbp)	0.355	0.04440
**27**	Dmbt1	deleted in malignant brain tumors 1(Dmbt1)	0.024	0.00005
**28**	Elovl3	elongation of very long chain fatty acids (FEN1/Elo2, SUR4/Elo3, yeast)-like 3(Elovl3)	2.786	0.00005
**29**	Fabp5	fatty acid binding protein 5, epidermal(Fabp5)	2.275	0.00005
**30**	Fam25c	family with sequence similarity 25, member C(Fam25c)	9.567	0.00005
**31**	Foxq1	forkhead box Q1(Foxq1)	2.324	0.00005
**32**	Gadd45g	growth arrest and DNA-damage-inducible 45 gamma(Gadd45g)	3.741	0.00005
**33**	Gas6	growth arrest specific 6(Gas6)	0.455	0.00005
**34**	Gm3219	B-cell CLL/lymphoma 7C pseudogene(Gm3219)	3.253	0.00005
**35**	Gstm2	glutathione S-transferase, mu 2(Gstm2)	0.430	0.00005
**36**	Gstm3	glutathione S-transferase, mu 3(Gstm3)	0.249	0.00005
**37**	H2-Eb1	histocompatibility 2, class II antigen E beta(H2-Eb1)	0.361	0.00005
**38**	Hopx	HOP homeobox(Hopx)	2.103	0.00005
**39**	Kcnk1	potassium channel, subfamily K, member 1(Kcnk1)	2.314	0.00005
**40**	Krt19	keratin 19(Krt19)	0.287	0.00005
**41**	Lpin1	lipin 1(Lpin1)	0.403	0.00005
**42**	Ly6a	lymphocyte antigen 6 complex, locus A(Ly6a)	0.172	0.00005
**43**	Ly6c1	lymphocyte antigen 6 complex, locus C1(Ly6c1)	0.166	0.00005
**44**	Ly6d	lymphocyte antigen 6 complex, locus D(Ly6d)	0.463	0.00160
**45**	Mfsd2a	major facilitator superfamily domain containing 2A(Mfsd2a)	0.352	0.00005
**46**	Moxd1	monooxygenase, DBH-like 1(Moxd1)	31.917	0.00005
**47**	Mup21	major urinary protein 21(Mup21)	2.180	0.00005
**48**	Myl9	myosin, light polypeptide 9, regulatory(Myl9)	0.405	0.00005
**49**	Rdh16	retinol dehydrogenase 16(Rdh16)	0.283	0.00005
**50**	Rdh9	retinol dehydrogenase 9(Rdh9)	0.350	0.00005
**51**	Rnaset2b	ribonuclease T2B(Rnaset2b)	0.175	0.00005
**52**	Saa3	serum amyloid A 3(Saa3)	0.448	0.00015
**53**	Scd1	stearoyl-Coenzyme A desaturase 1(Scd1)	0.290	0.00005

**54**	Serpina7	serine (or cysteine) peptidase inhibitor, clade A (alpha-1 antiproteinase, antitrypsin), member 7(Serpina7)	0.465	0.00005
**55**	Slc13a3	solute carrier family 13 (sodium-dependent dicarboxylate transporter), member 3(Slc13a3)	0.459	0.00005
**56**	Slc16a7	solute carrier family 16 (monocarboxylic acid transporters), member 7(Slc16a7)	0.487	0.00005
**57**	Snrnp25	small nuclear ribonucleoprotein 25 (U11/U12)(Snrnp25)	0.335	0.00005
**58**	Sult1a1	sulfotransferase family 1A, phenol-preferring, member 1(Sult1a1)	0.480	0.00005
**59**	Tceal8	transcription elongation factor A (SII)-like 8(Tceal8)	0.420	0.00005
**60**	Ubc	ubiquitin C(Ubc)	0.402	0.00005
**61**	Upp2	uridine phosphorylase 2(Upp2)	0.443	0.00005
**62**	Nlrp12	NLR family, pyrin domain containing 12	0.850	0.05117

Table S3. Compositions and Energy content of normal diet (AIN-93G) and High-fat diet (HFD45)

Formulations	AIN-93G		HFD 45%cal	
	g	kcal%	g	kcal%
Protein	20	20	24	20
Carbohydrate	64	64	41	35
Fat	7	16	24	45
kcal/kg	4,000		4,776	
**Ingredient**	**g**	**kcal**	**g**	**kcal**
Casein from milk	200	200	200	800
Corn starch	397.486	1590	155.036	620
Sucrose	100	400	50	200
Dextrose	132	528	132	528
Cellulose	50	0	50	0
Soybean Oil	70	630	25	225
Lard	0	0	175	1575
Minerals mixture	35	0	35	0
Vitamin mixture	10	40	10	40
TBHQ*	0.014	0	0.014	0
L-Cystine	3	15	3	12
Choline Bitartrate	2.5	0	2.5	0
